# Retraction Note: Prediction of DDoS attacks in agriculture 4.0 with the help of prairie dog optimization algorithm with IDSNet

**DOI:** 10.1038/s41598-026-60989-7

**Published:** 2026-07-06

**Authors:** Ramesh Vatambeti, D. Venkatesh, Gowtham Mamidisetti, Vijay Kumar Damera, M. Manohar, N. Sudhakar Yadav

**Affiliations:** 1 School of Computer Science and Engineering, VIT-AP University, Vijayawada, India; 2 Department of Computer Science and Engineering, GITAM School of Technology, GITAM University-Bengaluru Campus, Bengaluru, India; 3 Department of Computer Science and Engineering, Malla Reddy University, Hyderabad, India; 4 Department of Computer Science and Engineering, Koneru Lakshmaiah Education Foundation, Hyderabad, India; 5 Department of Computer Science and Engineering, CHRIST (Deemed to be University), Bangalore, India; 6 Department of Information Technology, Chaitanya Bharathi Institute of Technology, Hyderabad, 500075 India

Retraction of: *Scientific Reports* 10.1038/s41598-023-42678-x, published online 16 September 2023

The Editors have retracted this Article. After publication, concerns were raised about the non-standard terminology used in the article. Further investigation identified issues relating to the underlying data, and the code. The Authors were unable to provide an adequate explanation, or the code underlying this study. The Editors therefore no longer have confidence in the reliability of this Article.

Ramesh Vatambeti disagrees with this retraction. Venkatesh D, Gowtham Mamidisetti, Vijay Kumar Damera, Manohar M and N. Sudhakar Yadav did not respond directly to correspondence about this retraction.

